# Oral Vaccination Based on DNA-Chitosan Nanoparticles against *Schistosoma mansoni* Infection

**DOI:** 10.1100/2012/938457

**Published:** 2012-04-22

**Authors:** Carolina R. Oliveira, Cíntia M. F. Rezende, Marina R. Silva, Olga M. Borges, Ana P. Pêgo, Alfredo M. Goes

**Affiliations:** ^1^Departamento de Bioquímica e Imunologia, Instituto de Ciências Biológicas, Universidade Federal de Minas Gerais, Avenida Presidente Antônio Carlos 6627, ICB Q4-167, 31270-901 Belo Horizonte, MG, Brazil; ^2^Center for Neuroscience and Cell Biology (CNC), University of Coimbra, Largo Marquês de Pombal, 3004-517 Coimbra, Portugal; ^3^Faculdade de Farmácia, Universdade de Coimbra, Pólo das Ciências da Saúde, Azinhaga de Santa Comba, 3000-548 Coimbra, Portugal; ^4^Instituto de Engenharia Biomédica (INEB), Universidade do Porto, Rua do Campo Alegre 823, 4150-180 Porto, Portugal

## Abstract

The development of a vaccine would be essential for the control of schistosomiasis, which is recognized as the most important human helminth infection in terms of morbidity and mortality. A new approach of oral vaccination with DNA-chitosan nanoparticles appears interesting because of their great stability and the ease of target accessibility, besides chitosan immunostimulatory properties. Here we described that chitosan nanoparticles loaded with plasmid DNA encoding Rho1-GTPase protein of *Schistosoma mansoni*, prepared at different molar ratios of primary amines to DNA phosphate anion (N/P), were able to complex electrostatically with DNA and condense it into positively charged nanostructures. Nanoparticles were able to maintain zeta potential and size characteristics in media that simulate gastric (SGF) and intestinal fluids (SIF). Further *in vivo* studies showed that oral immunization was not able to induce high levels of specific antibodies but induced high levels of the modulatory cytokine IL-10. This resulted in a significative reduce of liver pathology, although it could not protect mice of infection challenge with *S. mansoni* worms. Mice immunized only with chitosan nanoparticles presented 47% of protection against parasite infection, suggesting an important role of chitosan in inducing a protective immune response against schistosomiasis, which will be more explored in further studies.

## 1. Introduction

Schistosomiasis is an important parasitic disease, caused by trematode worms of the genus Schistosoma, affecting more than 207 million people worldwide, with a further 700 million individuals living at risk of infection [1] and it causes up to 250,000 deaths per year [2]. Furthermore, the impact of the severe and debilitating effects of schistosomiasis accounts for the loss of 4.5 million disability adjusted life years (DALYs) annually [3].

Currently, schistosomiasis control strategies are predominantly based on the treatment of infected individuals with safe and effective drugs [4]. However, mass treatment has been proven to be insufficient to stop disease transmission, prevent reinfection, or reduce parasite-induced illness [5, 6]. Therefore a great effort to develop a protective vaccine to be used in combination with chemotherapy and improved sanitation in order to curb the menace of schistosomiasis is required.

Desirable characteristics of a schistosomiasis vaccine candidate include not only the capacity to reduce worm burden and fecundity but also the capacity to downregulate granulomatous responses to eggs that become trapped in the host liver and intestines and cause morbidity [7]. A vaccine that induces even a partial reduction in worm burdens could considerably reduce pathology and limit parasite transmission [8].

The identification of a specific antigen is a crucial task in the development of an effective vaccine. However, the antigens tested until now were found to induce insufficient levels of protection during the preclinical studies [9], what makes necessary the search for new antigens. In schistosomiasis, there is evidence indicating the involvement of low molecular weight proteins that bind to GTP in the process of maturation and deposition of eggs by the females of *S. mansoni* [10]. Therefore, the protein Rho1-GTPase of *S. mansoni* is probably related with the pathological response caused by parasites [11] and this brings an interest in understanding the role of this protein in immunological processes resulting from schistosomiasis and on the evaluation of its potential as a vaccine candidate.

Despite current challenges to improve delivery and immunogenicity, DNA vaccination has several major advantages over traditional vaccines or over other types of investigational vaccine platforms [12]. DNA vaccine technology is a simple concept based on relatively simple design and production technologies [13]. Another advantage of DNA vaccines over conventional protein vaccines is the low cost of production of a highly purified product.

To date, most gene delivery strategies have concentrated on the parenteral route of delivery and oral administration has been largely ignored. The main advantages presented by oral gene delivery are the ease of target accessibility and enhanced patient compliance owing to the noninvasive delivery method. For effective oral immunization, antigens and plasmids must be protected from the acidic and proteolytic environment of the gastrointestinal tract, efficiently taken up by cells of the gut associated lymphoid tissue (GALT), and an appropriate immune response must be induced. The interaction of plasmid DNA (pDNA) with a biodegradable cationic polymer to form nanoparticles offers a way to protect pDNA from degradation [14].

In this work, chitosan-based nanoparticles as adjuvant for mucosal vaccination were chosen. Chitosan (CH) has been considered an attractive gene carrier because it is nontoxic and biodegradable and has mucoadhesive properties. CH is able to form complexes easily with DNA [15, 16] which have been shown to effectively protect DNA from degradation [17]. In addition, CH has the capability to enhance the penetration of large molecules across the mucosal surface [14]. Finally, there has already been demonstrated CH particle's capability to be taken up by the Payer's patches [18] and its effectiveness as plasmid delivery systems to gut mucosal [19]. On the other hand, *in vitro* CH low transfection efficiency has so far hampered its widespread application. Therefore with the aim of improving CH gene delivery efficiency, CH has been functionalized with imidazole moieties, which acts to promote the endosomal escape capability of CH-DNA complexes [20]. In order to obtain higher rates of transfection, imidazole-grafted CH was used in the present work with the aim to prepare DNA-CH particles (encoding for Rho1-GTPase protein), for oral administration. Besides acting as a gene carrier, the use of CH to delivery DNA is even more attractive in this vaccination strategy considering his immunostimulatory characteristics, as has been largely described [21].

## 2. Materials and Methods

### 2.1. Materials

#### 2.1.1. Imidazole-Grafted CH

Imidazole-modified CH was prepared as previously described [20]. Briefly, technical grade CH (Chimarin, degree acetylation 13%, apparent viscosity 8 mPa.s, Medicarb, Sweden) was purified by filtration of an acidic CH solution and subsequent alkali precipitation (1 M NaOH). The purified polymer was characterized by gel permeation chromatography (GPC) and Fourier Transform-Infrared Spectroscopy (FT-IR). The average weight molecular weight of the starting material was found to be 1.2 × 10^5^ (GPC in 0.5 M CH_3_COOH—0.2 M CH_3_COONa, 25°C). The degree of acetylation determined by FT-IR according to Brugnerotto et al. [22] was found to be 16%. Endotoxin levels of the purified CH extracts were found to be lower than 0.1 EU/mL, respecting the US Department of Health and Human Services guidelines [23] for implantable devices. Imidazole-modified CH (CHimi) was obtained by the amidation of the glucosamine residues of CH using 1-ethyl-3-(3-dimethylaminopropyl) carbodiimide (EDC)/N-hydroxysuccinimide (NHS) condensation system. 22% of the primary amines of the original CH were substituted, as determined by FT-IR.

#### 2.1.2. Plasmid DNA

A plasmid (6176 pb), NT-GFP fusion TOPO TA (Invitrogen), containing Rho1-GTPase (1–579) cDNA fragment under the cytomegalovirus (CMV) early promoter was constructed previously in our laboratory, following the fabricant's recommendations. The cloning was verified by sequencing using GFP primers and confirmed the correct insertion of the Rho1-GTPase fragment in frame with GFP sequence.

The plasmid was amplified in DH5*α* *Escherichia coli *strand and isolated using the QIA-GEN Plasmid Maxi Kit (GeneElute HP, Sigma-Aldrich). The concentration and purity of the plasmid was accessed by spectrophotometric analysis. The ratio between optical density at 260 nm and 280 nm was >1.8 and <2.0.

#### 2.1.3. Recombinant Protein Rho1-GTPase of *S. mansoni*

Rho1-GTPase was expressed as His-fusion from pET-DEST42 (Invitrogen). The cloning was done using Gateway System (Invitrogen) and protein expression was carried out. The Rho1-GTPase was purified using the Ni-NTA His-Bind affinity chromatography. The His-Rho1-GTPase is hereafter referred to as SmRho.

### 2.2. Preparation of CH DNA Nanoparticles

Nanoparticles were prepared as previously described by mixing [20], while vortexing, equal volumes of polymer solution in 5 mM acetate buffer pH 5.5 (0.1% (w/v)) and plasmid DNA solution (in 25 mM Na_2_SO_4_), both previously heated at 55°C for 10 min. Complexes were allowed to form and stabilize for 15 min at RT before further use. Complexes with different polymer : DNA molar ratios were prepared. The ratio value (N/P molar ratio) is expressed in terms of moles of primary amine groups (N) of CH or CHimi to moles of DNA phosphate groups (P).

### 2.3. Characterization of Nanoparticles

#### 2.3.1. DNA Retardation Assay

Complexes were prepared at various N/P molar ratios as previously described; 2.5 *μ*g of plasmid DNA was used for the preparation of the nanoparticles in a final volume of 50 *μ*L and 20 *μ*L of each complex solution together with 4 *μ*L of loading buffer (Fermentas) was loaded in a 1% (w/v) agarose (Cambrex) gel, with 0.05 *μ*gmL^−1^ ethidium bromide (Q-BioGene). The electrophoresis was run at 90 V for 45 min, using Tris-acetate-EDTA buffer (pH 8) as the running buffer.

#### 2.3.2. Nanoparticles Size and Zeta Potential Determination

To characterize the size and zeta potential of the CH-DNA complexes as prepared, these were formed at different N/P molar ratios as described in Section 2.2 using 10 *μ*g of plasmid DNA. After stabilization, complexes were diluted to 1 mL, using 5 mM acetate buffer (pH 5.5). The zeta potential and mean hydrodynamic size of the nanoparticles were assessed using a Zetasizer Nano ZS (Malvern, UK). The Smoluchowski model was applied for determination of the zeta potential, and cumulant analysis was used for mean particle size determination. All measurements were performed in triplicate at 25°C.

To characterize the size and zeta potential of the nanoparticles when in the gastrointestinal tract conditions, the complexes were suspended in solutions that simulate the gastric (SGF) and intestinal fluid (SIF). For this purpose, complexes were prepared as described previously at N/P molar ratios of 12 and 18. After stabilization, complexes were diluted to 1 mL using either SGF or SIF (described in USP XXIV) at 37°C. After 15 min in these solutions, nanoparticles were characterized as described previously.

### 2.4. Immunization Studies

#### 2.4.1. Mice and Parasites

Six-week-old female C57BL6 mice were purchased from the Federal University of Minas Gerais (UFMG) animal facility. All protocols involving animals were approved by the local Ethics Committee on Animal Care (CETEA-UFMG number 204/2009). Animals had free access to food and water, with 12 h light/dark cycle.

*S. mansoni* cercariae were maintained routinely on Biomphalaria *glabrata* snails at the Center of Research Rene Rachou-Fiocuz (CPqRR). For infections, cercariae were prepared by exposing infected snails to light for 1 h to induce shedding. Cercariae numbers and viability were determined using a light microscope prior to infection.

#### 2.4.2. Treatment Groups

Five groups were submitted to different treatments, as follows:

Group I: control group—phosphate buffer saline (PBS) (*n* = 5);
Group II: suspension of CH nanoparticles (*n* = 5) administered by oral route;
Group III: solution of naked recombinant plasmid DNA (50 *μ*g) administered by oral route (*n* = 5);
Group IV: suspension of modified CH (CHimi)—plasmid DNA (25 *μ*g) nanoparticles administered by oral route (*n* = 5);
Group V: suspension of modified CH (CHimi)—plasmid DNA (25 *μ*g) nanoparticles administered by intramuscular route (*n* = 5).

In order to evaluate the adjuvant effect of CH, chitosan nanoparticles without DNA were prepared (Group II). And with the aim to evaluate the improvement of gene delivery by CH modified (Chimi), Chimi-DNA nanoparticles were administered to groups IV and V while group III received DNA without CH. The best route of administration was also analyzed comparing groups IV and V.

#### 2.4.3. Immunization Schedule and Collection of Samples

The different formulations were administered orally with a gavage-feeding needle (groups I–IV) and intramuscularly with an injection in the tibialis anterior muscle (group V). The primary immunization was followed by two immunizations with an interval of two weeks. Seven days after the last immunization mice were challenged and then after 50 days mice were sacrificed.

Blood samples were taken through tail veins from four mice of each experimental group at two-week intervals, and the sera were prepared by centrifugation and stored at −20°C until further analysis. Serum was collected from immunized and control mice to measure kinetics of specific antibodies.

### 2.5. Measurement of Specific Anti-SmRho Antibodies

A measurement of specific anti-SmRho antibodies was performed using an indirect enzyme-linked immunoabsorbent assay (ELISA). Maxisorp 96-well microtiter plates (Nunc, Roskilde, Denmark) were coated with 10 *μ*g/mL of SmRho in carbonate-bicarbonate buffer, pH 9.6, for 16 h at 4°C. Plates were washed with a solution containing PBS 0.05% Tween 20 (PBS-T) and were then blocked for 1 h at 37°C with 200 *μ*L/well of PBS-casein (phosphate buffer saline, pH 7.2 with 1.6% casein). Next, 100 *μ*L of each serum sample diluted 1 : 100 in PBS-casein (phosphate buffer saline, pH 7.2 with 0.25% casein) was added to each well and was incubated for 1 h at 37°C. Plate-bound antibodies were detected by peroxidase-conjugated anti-mouse IgG (SIGMA), IgG1, and IgG2a (Southern Biotechnology Associates, Inc., Birmingham, AL, USA) diluted 1 : 5000 in PBS with 0.25% casein. The plates were revealed by the addition of 100 *μ*L of detection solution (R&D systems, Minneapolis, USA) containing tetramethylbenzidine (Thermo Scientific Pierce) and H_2_O_2_ in each well; after 20 min reactions were stopped with the addition of 50 *μ*L of 5% (v/v) sulfuric acid per well. The absorbance was read at 450 nm in an ELISA plate reader (ELX 800 BIO-TEK Instruments Inc.).

### 2.6. Challenge Infection and Worm Burden Assessment

Seven days after the last immunization, mice were challenged through percutaneous exposure of abdominal skin to water containing 25 cercariae (LE strain) for one hour. 50 days after challenge, mice were sacrificed and adult worms were perfused from their portal veins [24, 25]. Protection was calculated by comparing the number of worms recovered from each vaccinated group compared with control group using (1):

(1) $$\text{PROTECTION}\;\left(\text{\%}\right)=\;\left[\frac{\left(\text{C}-\text{I}\right)}{\text{C}}\right]\times 100,$$

where C represents the worms recovered from the saline control group and I represents the worms recovered from the experimental group.

### 2.7. Cytokine Analysis

#### 2.7.1. Antigen Preparation

Antigenic preparations were obtained from schistosome eggs (SEA) and adult worms (SWAP), prepared as soluble supernatant fluids from buffered saline homogenates of the respective life-cycle stages [26]. Cytokine production was evaluated when splenocytes were stimulated with rSmRho, SWAP, or SEA.

#### 2.7.2. Cytokine Experiment

Cytokine experiments were performed using splenocyte cultures from individual mice of control and experimental groups. Splenocytes were isolated from macerated spleens of individual mice at day 50 post infection. Cells were washed twice with sterile PBS and were plated at a concentration of 1 × 10^6^ cells per well in RPMI 1640 medium (Gibco, Carlsbad, CA, USA) supplemented with 10% FBS, 100 U/mL of penicillin G sodium, 100 *μ*g/mL of streptomycin sulfate, and 250 ng/mL of amphotericin B. Splenocytes were maintained in culture with medium alone or stimulated with SmRho (25 *μ*g/mL), SWAP (25 *μ*g/mL), or SEA (25 *μ*g/mL). The 96-well plates (Nunc, Denmark) were maintained in an incubator at 37°C with 5% CO_2_. Culture supernatants were collected after 72 h for IL-10, IL-4, TGF-*β*, and IFN-*γ* analysis. IL-4, IL-10, IFN-*γ*, and TGF-*β* concentrations were measured using Duoset ELISA kit (R&D Systems, USA) according to the manufacturer's instructions.

### 2.8. Hepatic Granuloma Analysis

Liver fragments from mice (3 mice per group) of control and experimental groups immunized and infected were collected 50 days post infection in order to evaluate the effect of immunization on granuloma formation. Liver fragments were fixed in 10% paraformaldehyde. Fragments processed for paraffin embedding and histopathological sections were cut using a microtome at 5 *μ*m. Sections were stained on a slide with hematoxylin-eosin (HE). The areas of individual granulomas were obtained through the MacBiophotonics ImageJ software analyzer. Fifteen granulomas from each mouse with a single well-defined egg were randomly chosen using a microscope with the 4x objective lens; granulomas were then scanned using JVC TK-1270/RGB microcamera. Using a digital pad, the total area of each granuloma was measured, and the results were expressed in square micrometers (*μ*m^2^).

### 2.9. Statistical Analysis

Statistical analysis was performed using the ANOVA test and the GraphPad Prism 5 software package. The Bonferroni test was used to compare subgroups with the level of significance set at *P* < 0.05.

## 3. Results

### 3.1. Nanoparticles Characterization

**Figure 1:**
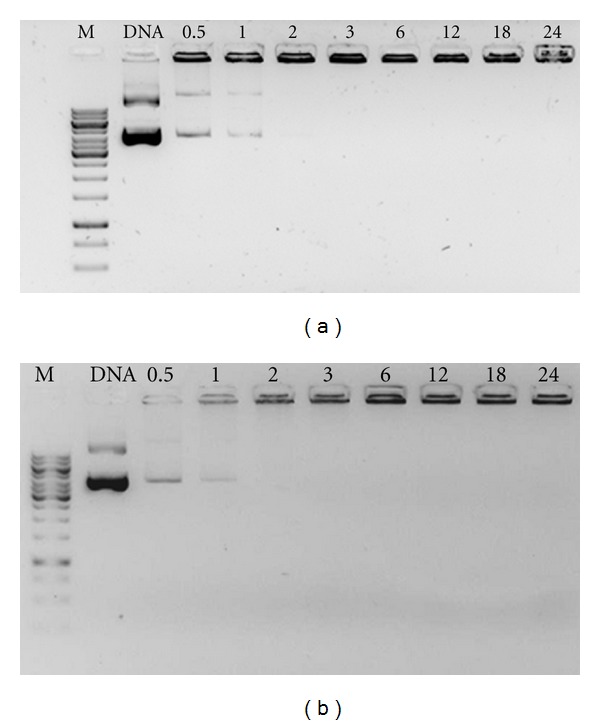
Electrophoretic retention of DNA by CH (a) and CHimi (b). Unless otherwise mentioned, lanes assignments correspond to N/P molar ratios tested and are as follows: Lane M: gene ruler 1 kb DNA ladder; lane DNA: plasmid DNA solution; lane 0.5–24: N/P molar ratios.

To determine the minimum amount of CHimi polymer required to complex plasmid DNA fully, varying amounts of polymer were mixed with DNA solutions with a fixed amount of plasmid DNA, and the resulting complexes were evaluated for their electrophoretic mobility. The modified polymers halt DNA mobility at the same N/P molar ratio as for nonmodified CH, which was found to be 2 (Figure 1).

**Figure 2:**
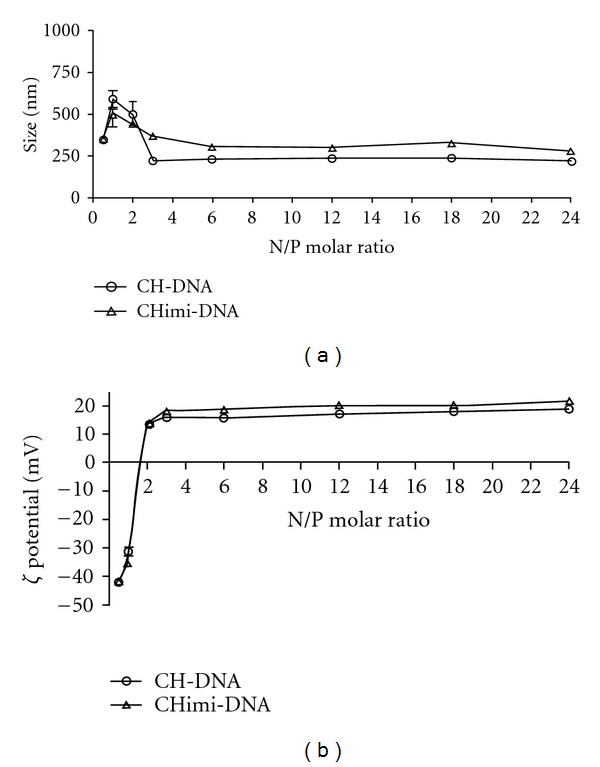
Mean particle size (a) and zeta potential (b) of CH-based complexes as a function of N/P molar ratio (measurements performed at 25°C, pH 5.5; average ± SD, *n* = 3).

The size of CH-DNA nanoparticles was determined by light scattering. Particles with N/P molar ratios raging from 1 to 24 were evaluated and results presented on Figure 2(a). For N/P molar ratios higher than 3, nanoparticles with a mean particle size around 300 nm were obtained independently of the polymer used. For lower N/P molar ratios, the size of the particles was around 500–600 nm.

Nanoparticles were also characterized in terms of zeta potential (Figure 2(b)). In both cases, zeta potential determinations have shown that an excess of polymer (N/P molar ratio >2) allowed the assembly of particles with a positive global net charge that stabilizes at a value between +16 mV and +20 mV, which is in agreement with the values found in the literature for complexes based on CH [17]. 

#### 3.1.1. Resistance of CH-DNA Nanoparticles to the Influence of pH and Temperature

**Figure 3:**
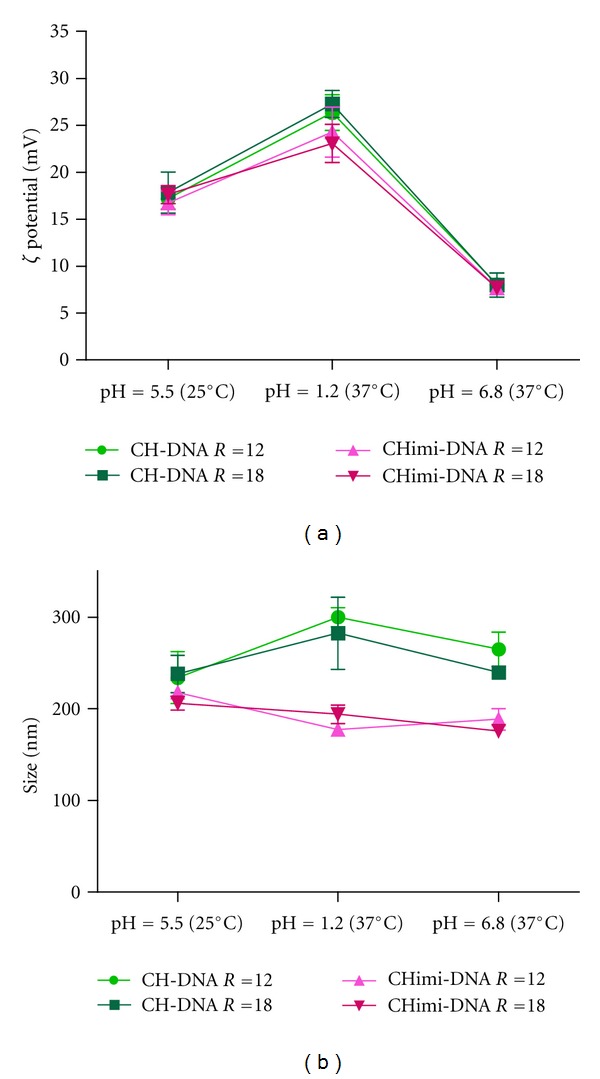
Zeta potential (a) and mean size (b) of CH-based complexes prepared in N/P molar ratios (R) 12 and 18 at different media. The measures were realized at 25°C in acetate buffer pH 5.5 and at a 37°C in SGF and SIF.

In order to evaluate the nanoparticles prepared as a candidate to oral delivery system for DNA vaccines, the stability of CH-DNA complexes in media that simulates the stomach and intestinal environment was tested. For this propose size and zeta potential of the particles suspended in SGF pH = 1.2 or SIF pH = 6.8, both at 37°C, were measured using nanoparticles with N/P molar ratios of 12 and 18. The results illustrated in Figure 3 show that the complexes were very stable at 37°C suspended in different buffers during the period of incubation. Small variations on size were observed but the range continues between 200 and 300 nm. On the other side, as predictable, the increase of the zeta potential to values near + 25 mV is explained by the higher protonation degree of the CH amine groups in acidic environment. Conversely, the contact with a higher pH solution resulted in the decrease of the zeta potential of the nanoparticles.

### 3.2. Antibody Profile following Mice Immunization

**Figure 4:**
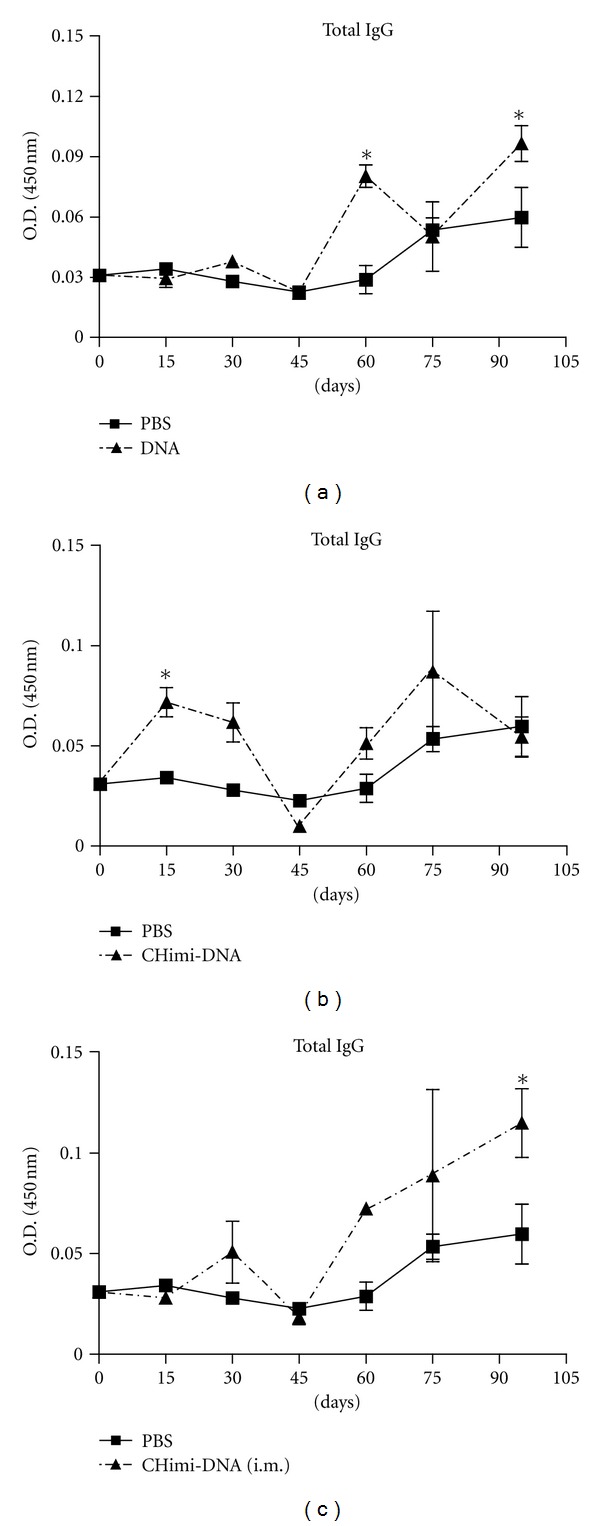
Serum anti-rSmRho-specific IgG levels of mice immunized orally with DNA (a) or CHimi-DNA (b) and intramusculary with CHimi-DNA nanoparticles (c). Sera of immunized mice were collected at days 15 (one week after the first immunization), 30, 45, 60, 75, and 95 and assayed by ELISA. The results are presented as the mean absorbance measured at 450 nm for each group. Statistically significant differences of vaccinated mice compared with the control group, PBS, in each time evaluated, are indicated by (*) for *P* < 0.05.

Further *in vivo* studies were performed to investigate the potential utility of oral nanoparticle-mediated gene immunization in eliciting the production of antibodies or modulating the immune response. To evaluate the levels of rSmRho-specific IgG antibodies serum samples from vaccinated animals from each group were tested by ELISA. The measures of IgG antibodies showed that nanoparticles were able to induce the production of specific rSmRho antibodies, even in small amounts. Significant levels of IgG antibodies could be verified in the serum collected from DNA-, CHimi-DNA-, and CHimi-DNA- (i.m.-) immunized groups, in determined times during the immunization or after the challenge infection (Figure 4). Although these levels were not sustained for long periods, it shows that the transfection was effective and allowed SmRho transcription by the DNA and CHimi-DNA nanoparticles. 

### 3.3. Protective Effect of CH-DNA Nanoparticles Vaccination

To investigate the protective activity induced by vaccination with CH and CHimi-DNA nanoparticles in murine model of *S. mansoni* infection, immunized mice were challenged with 25 cercariae. The difference in the number of adult worms recovered in the experimental groups compared to control group was calculated 50 days post challenge (Table 1). Only the group of animals immunized with CH nanoparticles without DNA and challenged with cercariae showed a significant reduction of 47% in adult worm burden, compared with the saline control group (mice received PBS) in (Table 1). This result suggests that CH has an important role in inducing protection against *S. mansoni* infection, and that the plasmid DNA, which codifies the SmRho protein, in this experiment, did not have a huge contribution in reducing worm burden. The later can be related to a lower rate of transfection and therefore the amount of protein expressed was not sufficient to give rise protective activity.

### 3.4. rSmRho Vaccination-Induced Granuloma Downmodulation

To evaluate the effect of the proposed vaccine on reducing granuloma reactions, histological analysis was performed by digital morphometry. Seven days after the third immunization, mice were challenged with 25 cercariae. After 50 days of challenge infection, mice were sacrificed and liver samples were taken for histological analysis. Hematoxylin and eosin stained liver sections were then used to measure the size of individual granulomas. CHimi-DNA nanoparticles vaccination by oral route reduced liver granuloma area by 37,0% (Table 1), compared with mice that were immunized with PBS (Table 1). Smaller granulomas were also observed in mice immunized with DNA without nanoparticles, with 24,9% of granuloma area reduction (Table 1). However these granulomas were not as small as those observed in the CHimi-DNA immunized groups. These findings suggest that the antigen codified by the plasmid DNA was important to induce this antipathological effect, and the better result observed in mice immunized CHimi-DNA nanoparticles is probably referred to the higher rate of transfection mediated by the modified CH (CHimi) compared with the group immunized with naked DNA. 

### 3.5. Cytokine Profiles Induced by rSmRho Immunization

**Figure 5:**
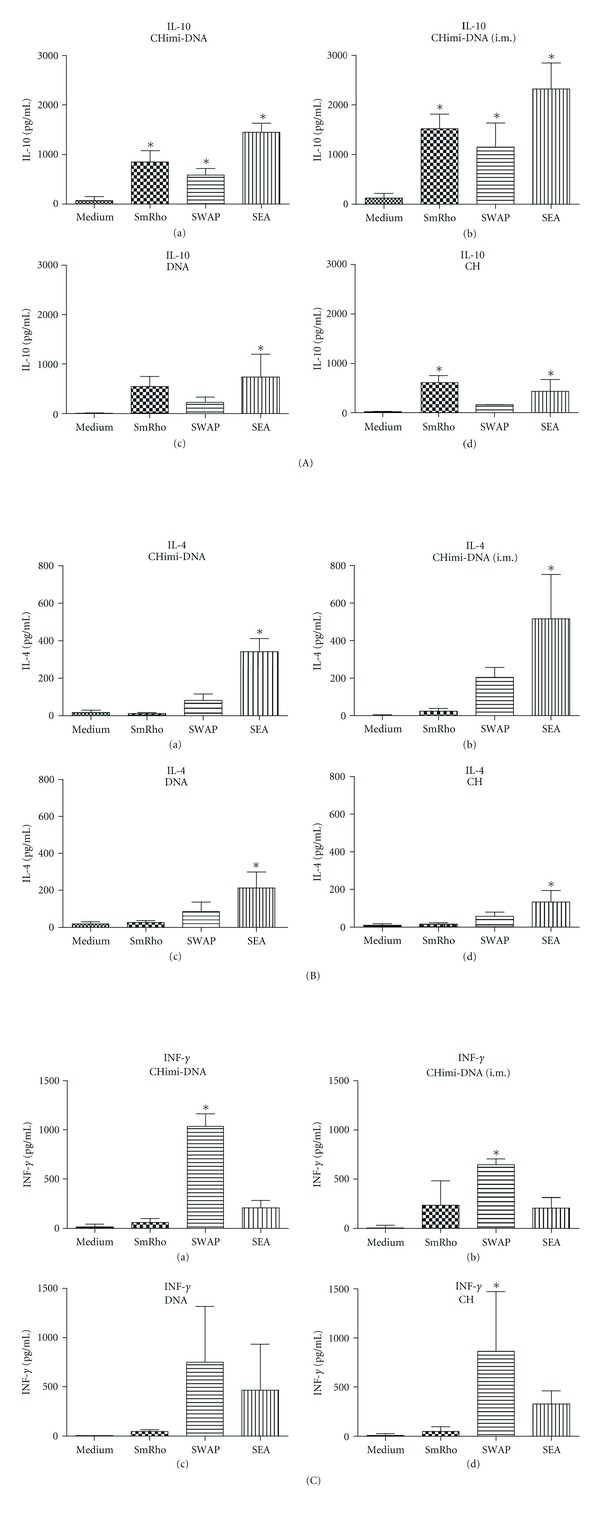
Cytokine profiles of mice immunized with CH-DNA nanoparticles. Splenocytes isolated from mice immunized with Chimi-DNA nanoparticles (a), CHimi-DNA (i.m.) (b), DNA (c), or CH (d) were assayed as IL-10 (A), IL-4 (B) and INF-*γ* (C), production in response to *in vitro* stimulation with SWAP (25 *μ*g/mL), rSmRho (25 *μ*g/mL), SEA (25 *μ*g/mL), or medium alone as control. Results represent the mean ± SD of each group. *Statistically significant differences between cytokines produced after SWAP, rSmRho, or SEA stimulation compared with unstimulated splenocytes (control) (*P* < 0.05).

Cytokine experiments were performed using splenocyte cultures from individual mice immunized with CHimi-DNA nanoparticles. Production (IFN-*γ*, IL-4, IL-10, and TGF-*β*) was measured in the supernatants of spleen cells cultured only in RPMI medium containing fetal bovine serum or in the presence of rSmRho, SWAP, or SEA. Higher levels of the immunomodulatory cytokine IL-10 were produced by rSmRho-stimulated splenocytes from CHimi-DNA-immunized group by intramuscular route and also from Chimi-DNA group immunized by oral route, compared with the control-stimulated splenocytes. Besides that, splenocytes from mice immunized with CH nanoparticles and stimulated with rSmRho showed a significative production of IL-10, although their levels were not so high compared with CHimi-DNA immunized groups (Figure 5 (A)). No significative levels of IFN-*γ*, a cytokine typical of Th1-type immune response, were produced by rSmRho-stimulated splenocytes from immunized groups, but when splenocytes from these groups were stimulated with SWAP, there were measured higher levels compared with the control-stimulated splenocytes (Figure 5 (B)). Over again no significant secretion of IL-4, a Th2 cytokine, was produced by rSmRho-stimulated splenocytes compared to control-stimulated splenocytes, but there was a considerable production of IL-4 when splenocytes of immunized groups were stimulated with SEA, what is a typical cytokine of granulomatous reaction (Figure 5 (C)). No significant difference on TGF-*β* production was detected between rSmRho, SWAP, or SEA-stimulated splenocytes from immunized groups (data not shown). These results indicate that the immunization of mice with those formulations of CHimi-DNA nanoparticles did not induce a predominant Th1 or Th2 immune response, but it induced mainly high levels of the immunomodulatory cytokine IL-10. 

## 4. Discussion 

Schistosomiasis is a chronic debilitating parasitic disease that represents a major health problem in endemic areas, such as various parts of South America, Africa, and Southeast Asia [27]. Since current schistosomiasis control programs applied to reduce morbidity and mortality have been proven inadequate and many commonly employed measures have been ineffective, there is a consensus that the development of an appropriate protective vaccine would be a crucial part of the overall integrated control strategy. 

In the present study, we found that it is possible to induce some arms of a protective immunity against experimental schistosomiasis in mice using CH and CHimi-DNA nanoparticles administered by oral route. This vaccination strategy offers many technical advantages, including a simple production process and the possibility of administration without the need of the use of needles, which facilitates administration mainly in development areas [28]. Additionally, polymeric carriers are potentially advantageous over viral vectors, which have *in vivo* limitations including wild type reversion and immunogenicity. The use of cationic lipids *in vivo* is also hindered by its toxicity, complement activation, and liver tropism. CH has been an attractive gene carrier because it is one of the few polycations of natural origin available and may be superior to other vectors including liposomes due to its minimal toxicity [29]. CH–DNA nanoparticles are spontaneously formed by the complex coacervation method using sodium sulphate as desolvating agent [17]. However, the efficacy presented by CH-based vectors is insufficient [30]. Moreira et al. [20] demonstrated that the introduction of imidazole moieties into the CH backbone leads to an improvement of its buffering capacity and promotes the endosomal escape capability of CH-DNA complexes via membrane destabilization of the acidified endosomes. It resulted in increasing the chances of reaching the nucleus and mediated a successful delivery of the transgene. In the present study, this modified CH (CHimi) was used with the aim of improving CH transfection efficiency. The characterization of nanoparticles showed that CH modified did not interfere with the basic properties of CH as described by Moreira et al. [20]. 

Electrophoretic retention of DNA was assessed for complexes prepared with increasing amounts of polymer. The modified polymer halts DNA mobility at the same N/P molar ratio (primary amines to plasmid DNA phosphate groups ratio) as nonmodified CH, which was found to be 2. Upon proving the ability of the modified polymers to complex DNA, it was important to evaluate their size and charge. For N/P molar ratios >3, no differences were observed in the behavior of CHimi-DNA or CH-DNA nanoparticles in terms of mean particle size and zeta potential. Large particles and polydisperse populations were observed only at lower N/P molar ratios. Other authors have also reported increased size at molar ratio 1 (the condition at which the zeta potential of the complexes is close to neutrality), which could be explained by the instability of the colloidal system. The dimension range referred to in the literature for CH-based complexes with DNA is quite wide and found to be strongly dependent on preparation conditions [17]. For particles prepared by the coacervation method, the size range reported was between 150 and 500 nm [17]. The results obtained with the present systems are therefore in close agreement with these findings. Besides that, complex size has an important role in uptake by cells. Smaller complexes have the advantage of entering in cells by endocytosis and/or pinocytosis, which favors, therefore, the efficiency of transfection [30]. In addition, the charge is also important to promote the uptake by cells, since many authors believe that particles charged positively favor their interactions with the cell membrane what facilitates their entrance in cells [31, 32]. 

To evaluate the behavior of these nanoparticles in media at different pHs and at physiological temperature, DNA-CH complexes were prepared at the N/P molar ratio of 12 and 18 and submitted to SGF pH = 1.2 and SIF pH = 6.8 to measure their size and zeta potential. The results showed that nanoparticles were resistant to pH media of SGF and SIF at 37°C. Complexes prepared with both CH and CHimi presented similar behavior, with little alterations in their size and zeta potential. To the best of our knowledge, this is the first time that such study has been conducted, showing that these nanoparticles will resist degradation during their passage in the gastrointestinal tract, what is desirable in oral immunizations. 

After characterization, the immunization with nanoparticles was realized to investigate the effect of CH-DNA nanoparticles by oral delivery on immune response in mice and their potential to treat the immunopathological responses or prevent infection. 

Our results demonstrated that the antipathological response induced against infection with cercariae is probably not related to the antibodies against rSmRho, because the assessment to the antibodies production by immunized mice showed low levels of rSmRho specific antibodies, although these levels had been significative at determined times during the period evaluated. On the other hand, the groups that showed the higher reduction in granuloma areas, CHimi-DNA (37%) and DNA (24.9%), were groups in which the formulation administered carried the plasmid DNA that codifies the SmRho protein. According to this, studies realized by other authors had suggested the involvement of this protein in process of maturation and egg deposition by *S. mansoni* females [10]. Besides that, Chen and Bennett [33] have related that the impairment of Rho1-GTPase synthesis can reduce egg production *in vitro* and *in vivo* by *S. mansoni* females, and therefore, it blocks the pathological effects of the disease in mice infected. These facts revealed an antipathological role of SmRho, but more studies are required to obtain a better understanding of the involvement of this protein in the process of infection of *S. mansoni*. 

The modulation of immunopathological responses of *S. mansoni* infection induced by SmRho is possibly related to the high levels of the regulatory cytokine IL-10 detected in splenocytes stimulation 

with rSmRho and also with SEA-stimulation. IL-10 plays a key regulatory role in facilitating the shift from a Th1 to Th2 response and preventing the development of severe pathology due to excessive Th1 and/or Th2 responses [34]. In the early stages of the granulomatous response, IL-10 acts to suppress the production of Th1 cytokines such as IFN-*γ* [35, 36], possibly by regulating IL-12/IL-12R expression which, in turn, modulates IFN-*γ* expression [37]. Hoffmann et al. [38] have also demonstrated that IL-10 is essential to prevent excessive Th2 responses in IL-10 knockout mice. It was the profile observed after DNA-nanoparticles immunization. 

To determine whether CHimi-DNA nanoparticles conferred protection against *S. mansoni *infection, immunized mice were challenged with cercariae and worm burdens were assessed. The higher percentage of reduction in worm burden was observed in mice immunized with CH nanoparticles without DNA, which was found to be 47%. Groups immunized with DNA or CHimi-DNA nanoparticles presented a considerable percentage of protection, which was 27% and 23%, respectively. The larger protection induced by CH suggests that it has an important role in stimulating the immune system and inducing a protective immune response against schistosomiasis. In fact, some authors have demonstrated that the major humoral immune responses in animals infected with *S. mansoni* are directed toward carbohydrate antigens. Among these antigens are complex-type N-glycans expressing LDN [GalNAcbeta1-4GlcNAc-R], LDNF [GalNAcbeta1-4(Fucalpha1-3)GlcNAc-R], and polymeric Lewis x (Lex) [Galbeta1-4(Fucalpha1-3)GlcNAc] n-R epitopes [39]. All these antigens have in common a structure of GlcNAc (*N*-Acetylglucosamine), and this monosaccharide is the monomeric unit of the polymer chitin, from which the CH derives from. Therefore, it could be suggested that the antibodies produced against the structure of CH can cross-react with these antigens present in the surface of parasites, and they have a role in conferring protection. Additionally, Nyame and coworkers [39] showed that monoclonal antibodies to LDN in the presence of complement efficiently kill schistosomula *in vitro*, what can be related in reduction of worm burden. However, to confirm this hypothesis further investigations are required. 

The lower rate of protection obtained by CHimi-DNA group can be explained by the number of CH nanoparticles in the formulation to be much less than the CH immunized group and also because the modified CH has a structure not so similar with the polysaccharides present in schistosoma tegument. 

Thus, we showed that the candidate of vaccine based on CHimi-DNA nanoparticles is able to modulate the granuloma area, which represents the major pathological response in schistosomiasis, and therefore, it can be useful in preventing or reducing such injuries of the disease, mainly in underdeveloped countries. Furthermore, the results obtained from these works, although being preliminaries data, suggest an important role of CH in inducing protection against infection of *S. mansoni*. Finally, we proposed that it is possible to merge both effects previously mentioned in an appropriate antischistosomiasis vaccine where the Chimi-DNA nanoparticles would be joined to CH nanoparticles, with the aim to obtain an effective protection and a reduced pathology in a single immunized group. 

## Figures and Tables

**Table 1:** Protective effect and liver granuloma size induced by C57BL/6 mice vaccination with CH-DNA nanoparticles and challenged with 25 *S. mansoni *cercariae.

Groups	Total worms mean ± SD	Percentage reduction of worm burden	Hepatic granuloma area (*μ*m^2^) Mean ± SD	Percentage reduction of granuloma area
Control	19.0 ± 2.4	—	109743 ± 47427	—
CH	10.1 ± 0.81*	47*	83269 ± 50226	24,1
DNA	13.8 ± 4.5	27	82352 ± 46222*	24,9*
CHimi-DNA	16.5 ± 3.6	23	69085 ± 40416*	37,0*
Chimi-DNA (i.m.)	17.5 ± 4.1	8	108222 ± 50046	1,39

*Statistically significant compared with the control group (*P* < 0.05).

## References

[2] Van Der Werf MJ, De Vlas SJ, Brooker S (2003). Quantification of clinical morbidity associated with schistosome infection in sub-Saharan Africa. *Acta Tropica*.

[3] Steinmann P, Keiser J, Bos R, Tanner M, Utzinger J (2006). Schistosomiasis and water resources development: systematic review, meta-analysis, and estimates of people at risk. *Lancet Infectious Diseases*.

[4] Harder A (2002). Chemotherapeutic approaches to schistosomes: current knowledge and outlook. *Parasitology Research*.

[5] Matthews KR (2011). Controlling and coordinating development in vector-transmitted parasites. *Science*.

[6] King CH (2009). Global health: toward the elimination of schistosomiasis. *New England Journal of Medicine*.

[7] Bergquist R (2004). Prospects for schistosomiasis vaccine development. *TDR News*.

[8] Chitsulo L, Loverde P, Engels D (2004). Schistosomiasis. *Nature Reviews Microbiology*.

[9] Oliveira SC, Fonseca CT, Cardoso FC, Farias LP, Leite LCC (2008). Recent advances in vaccine research against schistosomiasis in Brazil. *Acta Tropica*.

[10] Schüssler P, Grevelding CG, Kunz W (1997). Identification of Ras, MAP kinases, and a GAP protein in Schistosoma mansoni by immunoblotting and their putative involvement in male-female interaction. *Parasitology*.

[11] Loeffler IK, Bennett JL (1996). A rab-related GTP-binding protein in Schistosoma mansoni. *Molecular and Biochemical Parasitology*.

[12] Ledgerwood JE, Graham BS (2009). DNA vaccines: a safe and efficient platform technology for responding to emerging infectious diseases. *Human Vaccines*.

[13] Whalen RG (1996). DNA vaccines for emerging infectious diseases: what if?. *Emerging Infectious Diseases*.

[14] Bozkir A, Saka OM (2004). Chitosan nanoparticles for plasmid DNA delivery: effect of chitosan molecular structure on formulation and release characteristics. *Drug Delivery*.

[15] Singla AK, Chawla M (2001). Chitosan: some pharmaceutical and biological aspects—an update. *Journal of Pharmacy and Pharmacology*.

[16] Ranaldi G, Marigliano I, Vespignani I, Perozzi G, Sambuy Y (2002). The effect of chitosan and other polycations on tight junction permeability in the human intestinal Caco-2 cell line. *Journal of Nutritional Biochemistry*.

[17] Mao HQ, Roy K, Troung-Le VL (2001). Chitosan-DNA nanoparticles as gene carriers: synthesis, characterization and transfection efficiency. *Journal of Controlled Release*.

[18] Van Der Lubben IM, Verhoef JC, Van Aelst AC, Borchard G, Junginger HE (2001). Chitosan microparticles for oral vaccination: preparation, characterization and preliminary in vivo uptake studies in murine Peyer’s patches. *Biomaterials*.

[19] Roy K, Mao HQ, Huang SK, Leong KW (1999). Oral gene delivery with chitosan-DNA nanoparticles generates immunologic protection in a murine model of peanut allergy. *Nature Medicine*.

[20] Moreira C, Oliveira H, Pires LR, Simões S, Barbosa MA, Pêgo AP (2009). Improving chitosan-mediated gene transfer by the introduction of intracellular buffering moieties into the chitosan backbone. *Acta Biomaterialia*.

[21] Arca HC, Günbeyaz M, Şenel S (2009). Chitosan-based systems for the delivery of vaccine antigens. *Expert Review of Vaccines*.

[22] Brugnerotto J, Lizardi J, Goycoolea FM, Argüelles-Monal W, Desbrières J, Rinaudo M (2001). An infrared investigation in relation with chitin and chitosan characterization. *Polymer*.

[23] (2005). United States Pharmacopeia. *General Chapter 85—Bacterial Endotoxins Test*.

[24] Smithers SR, Terry RJ (1965). The infection of laboratory hosts with cercariae of Schistosoma mansoni and the recovery of the adult worms. *Parasitology*.

[25] Marques HH, Zouain CS, Torres CBB, Oliveira JS, Alves JB, Goes AM (2008). Protective effect and granuloma down-modulation promoted by RP44 antigen a fructose 1,6 bisphosphate aldolase of Schistosoma mansoni. *Immunobiology*.

[26] Goes AM, Rocha RS, Gazzinelli G, Doughty BL (1989). Production and characterization of human monoclonal antibodies against Schistosoma mansoni. *Parasite Immunology*.

[27] Schneider MC, Aguilera XP, da Silva Junior JB (2011). Elimination of neglected diseases in Latin America and the Caribbean: a mapping of selected diseases. *PLoS Neglected Tropical Diseases*.

[28] Kotton CN, Hohmann EL (2004). Enteric pathogens as vaccine vectors for foreign antigen delivery. *Infection and Immunity*.

[29] Illum L (1998). Chitosan and its use as a pharmaceutical excipient. *Pharmaceutical Research*.

[30] Mansouri S, Lavigne P, Corsi K, Benderdour M, Beaumont E, Fernandes JC (2004). Chitosan-DNA nanoparticles as non-viral vectors in gene therapy: strategies to improve transfection efficacy. *European Journal of Pharmaceutics and Biopharmaceutics*.

[31] Gao H, Shi W, Freund LB (2005). Mechanics of receptor-mediated endocytosis. *Proceedings of the National Academy of Sciences of the United States of America*.

[32] Chen L, Mccrate JM, Lee JC-M, Li H (2011). The role of surface charge on the uptake and biocompatibility of hydroxyapatite nanoparticles with osteoblast cells. *Nanotechnology*.

[33] Chen GZ, Bennett JL (1993). Characterization of mevalonate-labeled lipids isolated from parasite proteins in Schistosoma mansoni. *Molecular and Biochemical Parasitology*.

[34] Wilson MS, Mentink-Kane MM, Pesce JT, Ramalingam TR, Thompson R, Wynn TA (2007). Immunopathology of schistosomiasis. *Immunology and Cell Biology*.

[35] Hesse M, Piccirillo CA, Belkaid Y (2004). The pathogenesis of Schistosomiasis is controlled by cooperating IL-10-producing innate effector and regulatory T cells. *Journal of Immunology*.

[36] Wynn TA, Cheever AW, Williams ME (1998). IL-10 regulates liver pathology in acute murine Schistosomiasis mansoni but is not required for immune down-modulation of chronic disease. *Journal of Immunology*.

[37] Todt JC, Whitfield JR, Ivard SR, Boros DL (2000). Down-regulation of interleukin-12, interleukin-12r expression/ activity mediates the switch from Th1 to Th2 granuloma response during murine Schistosomiasis mansoni. *Scandinavian Journal of Immunology*.

[38] Hoffmann KF, Cheever AW, Wynn TA (2000). IL-10 and the dangers of immune polarization: excessive type 1 and type 2 cytokine responses induce distinct forms of lethal immunopathology in murine schistosomiasis. *Journal of Immunology*.

[39] Nyame AK, Lewis FA, Doughty BL, Correa-Oliveira R, Cummings RD (2003). Immunity to schistosomiasis: glycans are potential antigenic targets for immune intervention. *Experimental Parasitology*.

